# Mortality Related to Chagas Disease and HIV/AIDS Coinfection in Brazil

**DOI:** 10.1155/2012/534649

**Published:** 2012-08-28

**Authors:** Francisco Rogerlândio Martins-Melo, Alberto Novaes Ramos, Carlos Henrique Alencar, Jorg Heukelbach

**Affiliations:** ^1^Department of Community Health, School of Medicine, Federal University of Ceará, 60430-140 Fortaleza, CE, Brazil; ^2^Swiss Tropical and Public Health Institute, University of Basel, 4002 Basel, Switzerland; ^3^Anton Breinl Centre for Public Health and Tropical Medicine, School of Public Health, Tropical Medicine and Rehabilitation Sciences, James Cook University, Townsville, QLD 4811, Australia

## Abstract

Chagas disease in patients with HIV infection represents a potentially serious event with high case fatality rates. This study describes epidemiological and clinical aspects of deaths related to Chagas disease and HIV/AIDS coinfection in Brazil, 1999–2007. We performed a descriptive study based on mortality data from the nationwide Mortality Information System. Of a total of about 9 million deaths, Chagas disease and HIV/AIDS were mentioned in the same death certificate in 74 cases. AIDS was an underlying cause in 77.0% (57) and Chagas disease in 17.6% (13). Males (51.4%), white skin color (50%), age group 40–49 years (29.7%), and residents in the Southeast region (75.7%) were most common. Mean age at death was significantly lower in the coinfected (47.1 years [SD ± 14.6]), as compared to Chagas disease deaths (64.1 years [SD ± 14.7], *P* < 0.001). Considering the lack of data on morbidity related to Chagas disease and AIDS coinfection, the use of mortality data may be an appropriate sentinel approach to monitor the occurrence of this association. Due to the epidemiological transition in Brazil, chronic Chagas disease and HIV/AIDS coinfection will be further complicated and require the development of evidence-based preventive control measures.

## 1. Introduction 

Chagas disease, caused by the protozoan parasite *Trypanosoma cruzi*, is a well-known opportunistic infection in people living with HIV/AIDS [1–6]. Reactivation of chronic indeterminate Chagas disease in patients with HIV infection represents a serious event with high case fatality rates [1, 3, 4]. New aspects of the immunopathology of Chagas disease have been described recently in patients infected with HIV, and unusual clinical manifestations such as skin lesions, involvement of the central nervous system (meningoencephalitis), and/or serious heart damage (myocarditis) related to the reactivation of the disease have been reported [2–4].

The first case of HIV/*T. cruzi *coinfection was reported in the 1980s, but data on several issues are still scanty, such as the frequency of its occurrence, clinical and laboratorial profile of subjects with coinfection, survival rates, and mortality [1, 7, 8]. 

Chagas disease is endemic in 21 Latin American countries. Due to migration of Latin Americans, an increasing public health impact has been observed in nonendemic countries, such as in Australia, Canada, Japan, Spain, and the United States [5–7, 9]. Thus, the overlap of HIV infection and *T. cruzi* may occur not only in endemic areas, but also in wealthier regions that receive an increasing number of potentially infected migrants [1, 6]. 

Despite the relevance, the clinical importance of this coinfection and its epidemiology is unknown in Brazil and other endemic countries [1]. Here, we present an analysis of deaths related to Chagas disease and HIV/AIDS coinfection in Brazil, based on multiple causes of death.

## 2. Materials and Methods

### 2.1. Study Design and Population

We performed a descriptive study on population-based nationwide mortality data, obtained from the Brazilian Mortality Information System (*SIM—Sistema de Informação sobre Mortalidade*). *SIM* data sets are based on the death certificates (*Declaração de óbito*), consisting of standardized forms to be filled out by the physicians in charge. Death certificates contain demographic data (age, gender, education, race, marital status, municipality of residence, and municipality of occurrence of death) and clinical information (underlying and associated causes of death).

*SIM* data are public domain and freely available at the website of the Informatics Department of Unified Health System (*DATASUS*, http://tabnet.datasus.gov.br/cgi/deftohtm.exe?sim/cnv/obt10uf.def).

We included deaths that occurred in Brazil between 1999 and 2007, in which Chagas disease and AIDS were mentioned in the same Death Certificate, both as underlying or associated cause of death (so-called multiple causes of death).

### 2.2. Data Processing and Analysis

Downloading of data sets and data processing has been described in detail previously [10]. Briefly, a total of 243 mortality data sets with about 9 million entries were downloaded. We obtained the study population of coinfected individuals by selecting data sets where Chagas disease and HIV/AIDS were mentioned in any field of death certificates. Available demographic and clinical data were used to characterize the study population. We described frequencies and proportions by gender, age, race, region of residence, residence and occurrence in state capital, and year of death.

Chagas disease as a cause of death corresponded to the category B57 (Chagas disease), including all clinical forms of the Tenth Revision of the International Statistical Classification of Diseases and Related Health Problems (ICD-10) [11]. HIV/AIDS as a cause of death was identified in ICD-10 by the group B20-B24 (disease by human immunodeficiency virus—HIV) [11].

In addition, we present clinical forms of Chagas disease and HIV/AIDS that were associated with coinfection. The description was performed by disease or disorders coded according to ICD-10. Individual death certificates may have more than one clinical form of Chagas disease or AIDS.

Data were stored and analysed by STATA version 11 (Stata Corporation, College Station, TX, USA).

### 2.3. Ethics

This study was solely based on publicly available secondary anonymous data, with no possibility of identification of individuals. Thus, approval by an ethical review board was not necessary.

## 3. Results

Between 1999 and 2007, a total of 8,942,217 deaths occurred in Brazil, with 53,930 (0.6%) deaths related to Chagas disease, and 103,075 (1.1%) related to HIV/AIDS. We identified 74 deaths in which Chagas disease and HIV/AIDS were mentioned on the same Death Certificate, either as underlying or associated causes of death. 

Of these coinfected cases, AIDS was an underlying cause of death in 57 (77.0%), while Chagas disease was listed in 13 (17.6%). Chagas disease and HIV/AIDS were presented as associated causes in four deaths with other underlying causes: liver cancer (2), acute myocardial infarction (1), and skin abscess (1). 

Table 1 depicts epidemiological characteristics of deaths related to Chagas disease and HIV/AIDS coinfection. Males (51.4%), whites (50%), 40–49 year olds (29.7%), and residents of the Southeast region (75.7%) were most common. The mean age at death was significantly lower in the coinfected (47.1 years [SD ± 14.6]), as compared to the total number of Chagas disease deaths (as published previously [10]: 64.1 years [SD ± 14.7], *P* < 0.001).

Clinical manifestations of HIV/AIDS included other infectious and parasitic diseases (ICD-10: B20.8) (37.8%) and unspecified HIV disease (B24) (29.7%; Table 2). In Chagas disease, the chronic cardiac forms (B57.2) were predominant (77%; Table 2). Acute Chagas disease with cardiac involvement (B57.0) (8.1%) and chronic Chagas disease affecting the nervous system (B57.4) (6.8%; Table 2) were more common among the HIV-infected as compared to all deaths by Chagas disease (2.5% and 0.3%, resp.).

## 4. Discussion

This is the first national population-based analysis of Brazilian mortality data related to Chagas disease and HIV/AIDS coinfection. In fact, *T. cruzi*/HIV coinfection has not been systematically evaluated in the majority of endemic countries for Chagas disease [7]. The data show an association of coinfection with early mortality, compared to deaths from Chagas disease only, as described in a previous study [10]. The magnitude of both AIDS and Chagas disease in Brazil as chronic conditions will probably increase the likelihood of occurrence of coinfection in the future [12]. 

The epidemiology of Chagas disease has changed in recent decades, with a shift to older age groups, as a consequence of the control of its main vector (the kissing bug *Triatoma infestans*) and the control of transmission by blood transfusion [1, 10]. The control of these main means of transmission of Chagas disease may have caused this observed higher frequency of deaths in the chronic phase [10, 13]. Our study shows that in contrast to this trend deaths from coinfection were found predominantly in young adults. Our observation is consistent with the epidemiological profile of coinfected subjects described in previous studies: adult males from endemic regions, with serological diagnosis in the indeterminate form of the chronic phase and reactivation of Chagas disease [7].

The lower survival rate of subjects with coinfection is related to reactivation of Chagas disease and complications of both diseases [7]. Myocarditis and meningoencephalitis played also an important role in coinfection deaths as compared to Chagas disease. This indicates that Chagas reactivation in the central nervous system and myocardium is usually severe, often with fatal results [7]. Reactivation is suspected when the coinfected subject presents clinically acute Chagas disease or clinical decompensation of the chronic phase, organic impairment uncommon in Chagas disease, or pseudotumoral brain lesions [5, 7, 12]. In the case of absent or controlled reactivation, survival is directly related to the complications of Chagas disease and of HIV/AIDS infection. In the case of central nervous system involvement, delay in diagnosis of Chagas neurological damage and late introduction of specific therapy against *T. cruzi* increases case fatality [3, 8]. However, predictive factors for reactivation of Chagas disease are not yet fully understood [7, 14].

Reactivation of Chagas disease has been recognized as an opportunistic disease and was included as an AIDS-defining event in Brazil in 2003 [15]. Brazil has developed since 2006 a National Network of Attention and Studies in *T. cruzi*/HIV coinfection that currently involves cooperation with other countries, like Argentina and Spain [1].

Overlapping of HIV and *T. cruzi* infections also occurs in nonendemic areas of North America and Europe. The implementation of screening programs for migrant populations is necessary for early diagnosis of Chagas disease [6, 9].

Due to the lack of systematic data on morbidity related to Chagas disease and AIDS coinfection, the use of mortality data may be an appropriate sentinel approach to monitor the occurrence of this association. Mortality data can be considered as valid in Brazil, as they are well recorded in DATASUS database and undergo quality control [16, 17]. Limitations of the study may include problems arising from disease notification and data entry [16], and secondary data may have shown inconsistencies in the quantity and quality of information [17]. Deaths may be underreported, despite the progress made during the observation period in terms of *SIM *coverage and quality of information on causes of deaths. The coverage (ratio of deaths reported/estimated) also presents variations between regions in the country, with lower coverage mainly in the North and Northeast Regions [17]. The results of this study show internal consistency and coherence with existing knowledge about Chagas disease and HIV/AIDS. 

We consider data as highly representative, since all death certificates during the period 1999 to 2007 were included, in a country of continental dimensions.

## 5. Conclusions

The use of multiple causes of death allowed to describe the magnitude and epidemiological characteristics of mortality related to Chagas disease and HIV/AIDS coinfection in Brazil. Due to the ongoing epidemiological transition from the predominance of infectious diseases to more chronic and lifestyle-related ones in Brazil, chronic Chagas disease and HIV/AIDS coinfection require comprehensive and reliable information that supports the development of preventive control measures. There is a clear demand for comprehensive care from primary service providers to reference centers and to structure a network of comprehensive care to deal with this situation, with the mobilization that goes from primary care to highest level of technological complexity.

## Figures and Tables

**Table 1:** Sociodemographic characteristics of deaths related to Chagas disease and HIV/AIDS co-infection in Brazil, from 1999 to 2007 (*n* = 74).

Characteristics	*N*	%
Sex		
Male	38	51.4
Female	36	48.6
Age group (years)		
<15	2	2.7
15–29	4	5.4
30–39	18	24.3
40–49	14	18.9
50–59	22	29.7
60–69	11	14.9
≥70	3	4.1
Age at death		
<50	38	51.4
≥50	36	48.6
Race/color		
Caucasian	37	50.0
Brown	18	24.3
Black	11	14.9
Ignored	8	10.8
Region of residence in Brazil		
Southeast	56	75.7
Central West	10	13.5
Northeast	4	5.4
South	4	5.4
Residence in state capital		
No	51	68.9
Yes	23	31.1
Death in state capital		
Yes	39	52.7
No	35	47.3

**Table 2:** Distribution of deaths related to Chagas disease and HIV/AIDS, according to clinical presentation, Brazil, from 1999 to 2007 (*n* = 74).

Clinical form (ICD-10)	*N*	%
HIV/AIDS*		
HIV disease resulting in other infectious and parasitic diseases (B20.8)	28	37.8
Unspecified human immunodeficiency virus [HIV] disease (B24)	22	29.7
HIV disease resulting in multiple infections (B20.7)	18	24.3
HIV disease resulting in other bacterial infections (B20.1)	3	4.1
HIV disease resulting in multiple diseases classified elsewhere (B22.7)	2	2.7
HIV disease resulting in mycobacterial infection (B20.0)	1	1.4
HIV disease resulting in other specified conditions (B23.8)	1	1.4
Chagas disease**		
Chagas disease (chronic) with heart involvement (B57.2)	57	77.0
Acute Chagas disease with heart involvement (B57.0)	6	8.1
Acute Chagas disease without heart involvement (B57.1)	5	6.8
Chagas disease (chronic) with nervous system involvement (B57.4)	5	6.8
Chagas disease (chronic) with digestive system involvement (B57.3)	2	2.7
Chagas disease (chronic) with other organ involvement (B57.5)	1	1.4

*In one case two clinical forms were presented.

**In two cases two clinical forms were presented.
